# Supplementary material for: Inferring the role of the microbiome on survival in patients treated with immune checkpoint inhibitors: causal modeling, timing, and classes of concomitant medications
Source: BMC Cancer. 2020 May 6;20:383. doi: 10.1186/s12885-020-06882-6 (PMC7201618; doi:10.1186/s12885-020-06882-6)

Figure S1: Causal Model with references

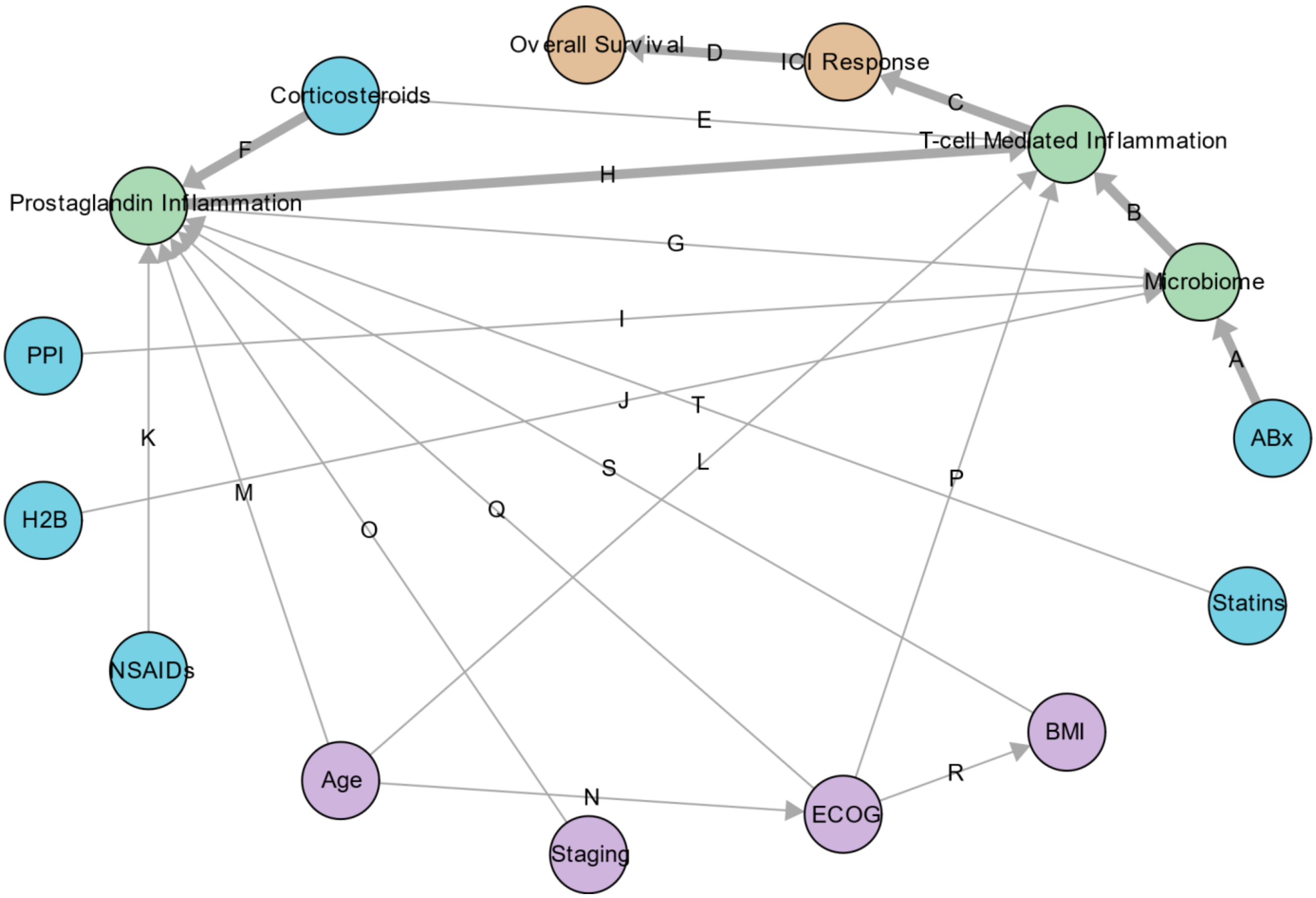

Table of references for each link:

Edge Label	Interaction	References
A	ABx > Microbiome	1-4
B	Microbiome > T-cell Mediated Inflammation	1,4,5
C	T –Cell Mediated Inflammation > ICI response	6
D	ICI response > OS	1,4,5
E	CS >T-Cell Mediated Inflammation	7
F	CS > Prostaglandin Inflammation	7
G	Prostaglandin Inflammation > Microbiome	8
H	Prostaglandin Inflammation > T-Cell Mediated Inflammation	9
I	PPI > Microbiome	10-13
J	H2B > Microbiome	13
K	NSAID > Prostaglandin Inflammation	9
L	Age > T-Cell Mediated Inflammation	14
M	Age > Prostaglandin Inflammation	15
N	Age > ECOG	16
O	Staging > Prostaglandin Inflammation	9
P	ECOG > T-Cell Mediated Inflammation	17

Q	ECOG > Prostaglandin Inflammation	17,18
R	ECOG > BMI	19
S	ECOG > Prostaglandin Inflammation	18
T	Statins > Prostaglandin Inflammation	20

Reference key:

1. Routy B, Le Chatelier E, Derosa L, Duong CP, Alou MT, Daillère R, et al. Gut microbiome influences efficacy of PD-1–based immunotherapy against epithelial tumors. Science. 2018;359(6371):91–97.

2. Dethlefsen L, Relman DA. Incomplete recovery and individualized responses of the human distal gut microbiota to repeated antibiotic perturbation. Proceedings of the National Academy of Sciences. 2011 Mar 15;108(Supplement_1):4554–61.

3. Boursi B, Mamtani R, Haynes K, Yang Y-X. Recurrent antibiotic exposure may promote cancer formation – Another step in understanding the role of the human microbiota? European Journal of Cancer. 2015 Nov;51(17):2655–64.

4. Matson V, Fessler J, Bao R, Chongsuwat T, Zha Y, Alegre M-L, et al. The commensal microbiome is associated with anti–PD-1 efficacy in metastatic melanoma patients. Science. 2018 Jan 5;359(6371):104–8.

5. Gopalakrishnan V, Spencer CN, Nezi L, Reuben A, Andrews MC, Karpinets TV, et al. Gut microbiome modulates response to anti–PD-1 immunotherapy in melanoma patients. Science. 2018 Jan 5;359(6371):97–103.

6. Chheda ZS, Sharma RK, Jala VR, Luster AD, Haribabu B. Chemoattractant Receptors BLT1 and CXCR3 Regulate Antitumor Immunity by Facilitating CD8^{+} T Cell Migration into Tumors. JI. 2016 Sep 1;197(5):2016–26.

7. Arbour KC, Mezquita L, Long N, Rizvi H, Auclin E, Ni A, et al. Impact of Baseline Steroids on Efficacy of Programmed Cell Death-1 and Programmed Death-Ligand 1 Blockade in Patients With Non–Small-Cell Lung Cancer. JCO. 2018 Oct;36(28):2872–8.

8. Wu T, Yang L, Jiang J, Ni Y, Zhu J, Zheng X, et al. Chronic glucocorticoid treatment induced circadian clock disorder leads to lipid metabolism and gut microbiota alterations in rats. Life Sciences. 2018 Jan;192:173–82.

9. Hussain M, Javeed A, Ashraf M, Al-Zaubai N, Stewart A, Mukhtar MM. Non-steroidal anti-inflammatory drugs, tumour immunity and immunotherapy. Pharmacological Research. 2012 Jul;66(1):7–18.

10. Maier L, Pruteanu M, Kuhn M, Zeller G, Telzerow A, Anderson EE, et al. Extensive impact of non-antibiotic drugs on human gut bacteria. Nature. 2018 Mar;555(7698):623–8.

11. Imhann F, Bonder MJ, Vich Vila A, Fu J, Mujagic Z, Vork L, et al. Proton pump inhibitors affect the gut microbiome. Gut. 2016 May;65(5):740–8.

12. Jackson MA, Goodrich JK, Maxan M-E, Freedberg DE, Abrams JA, Poole AC, et al. Proton pump inhibitors alter the composition of the gut microbiota. Gut. 2016 May;65(5):749–56.

13. Rogers MAM, Aronoff DM. The influence of non-steroidal anti-inflammatory drugs on the gut microbiome. Clinical Microbiology and Infection. 2016 Feb 1;22(2):178.e1-178.e9.

14. Fülöp T, Larbi A, Pawelec G. Human T Cell Aging and the Impact of Persistent Viral Infections. Front Immunol [Internet]. 2013 Sep 13 [cited 2019 Jul 24];4. Available from: https://www.ncbi.nlm.nih.gov/pmc/articles/PMC3772506/

15. Goodwin JS, Messner RP. Sensitivity of lymphocytes to prostaglandin E2 increases in subjects over age 70. J Clin Invest. 1979 Aug 1;64(2):434–9.

16. Hickey ME, Mason SE. Age and gender differences in particpation rates, motivators for, and barriers to exercise. :11.

17. Pedersen BK, Hoffman-Goetz L. Exercise and the Immune System: Regulation, Integration, and Adaptation. Physiological Reviews. 2000 Jul 1;80(3):1055–81.

18. Martínez ME, Heddens D, Earnest DL, Bogert CL, Roe D, Einspahr J, et al. Physical Activity, Body Mass Index, and Prostaglandin E 2 Levels in Rectal Mucosa. J Natl Cancer Inst. 1999 Jun 2;91(11):950–3.

19. Fang J, Wylie-Rosett J, Cohen HW, Kaplan RC, Alderman MH. Exercise, body mass index, caloric intake, and cardiovascular mortality. American Journal of Preventive Medicine. 2003 Nov 1;25(4):283–9.

20. Levine L. Statins stimulate arachidonic acid release and prostaglandin I2 production in rat liver cells. Lipids Health Dis. 2003 Mar 12;2:1.

Figure S2. Number of antibiotics, separated by class, prescribed across all cancers and the frequency of multiple antibiotics.

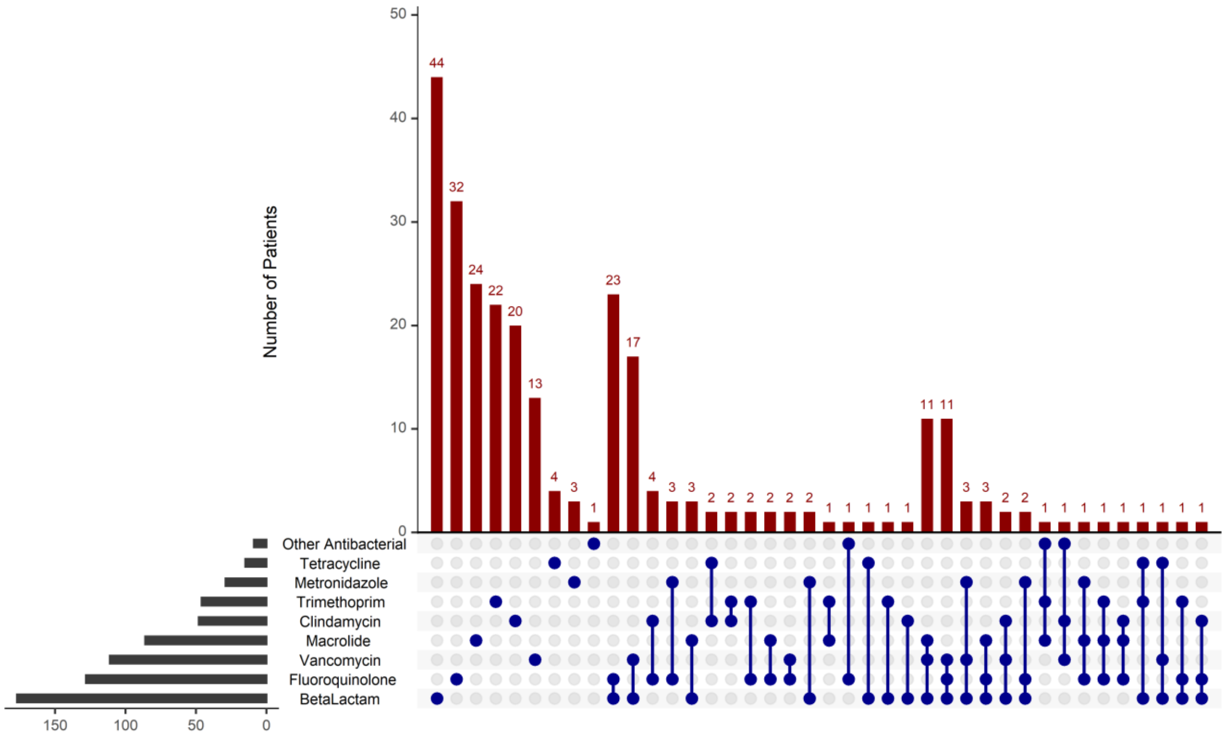

Figure S3. Number of corticosteroids, separated by class, prescribed across all cancers and the frequency of multiple antibiotics.

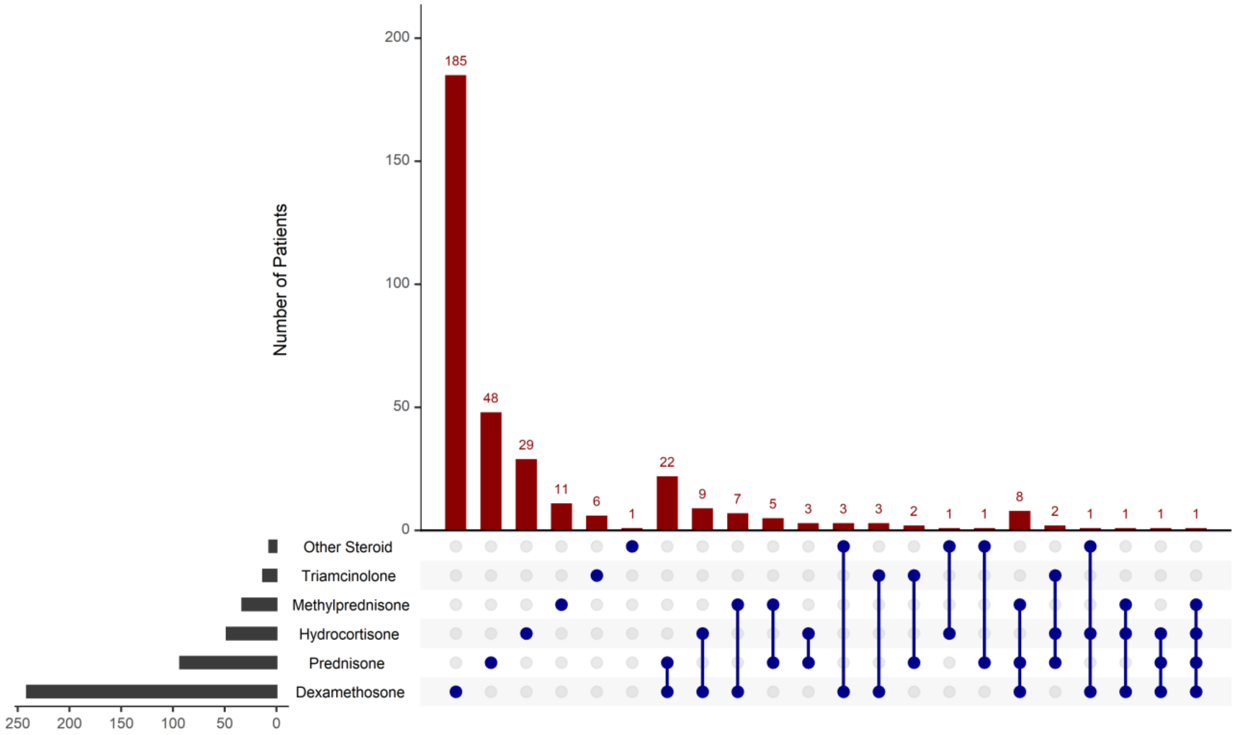

Figure S4. Number of medications prescribed across all cancers and the frequency of multiple medications.

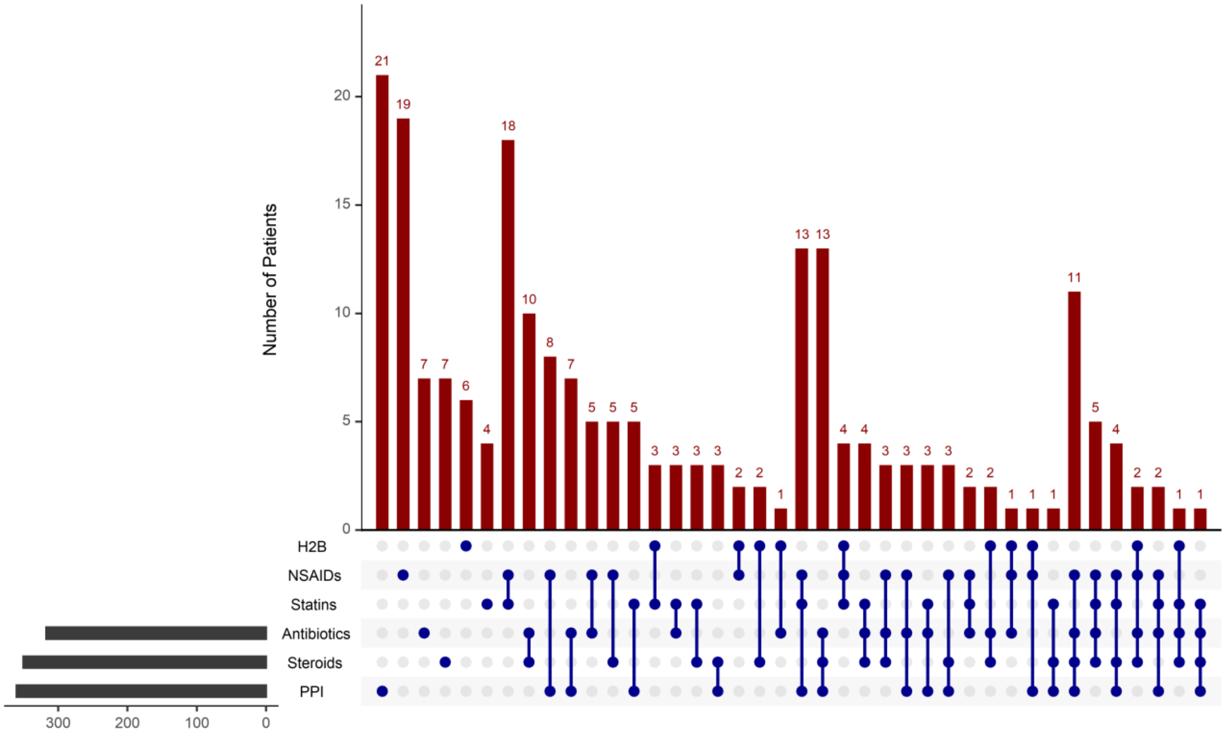

Supplement: Supplementary file 1 — Additional file 1: Figure S1. Causal diagram with references. Figure S2. Number of medications prescribed across all cancers and the frequency of multiple medications. Figure S3. Number of antibiotics, separated by class, prescribed across all cancers and the frequency of multiple antibiotics. Figure S4. Number of corticosteroids, separated by class, prescribed across all cancers and the frequency of multiple antibiotics. [file 12885_2020_6882_MOESM1_ESM.pdf]
