# Supplementary material for: Inferring the role of the microbiome on survival in patients treated with immune checkpoint inhibitors: causal modeling, timing, and classes of concomitant medications
Source: BMC Cancer. 2020 May 6;20:383. doi: 10.1186/s12885-020-06882-6 (PMC7201618; doi:10.1186/s12885-020-06882-6)
Supplement: Supplementary file 2 — Additional file 2: Table S1. References for the antibiotic susceptibilities of bacterial taxa found to be enriched in responders or non-responders to ICI therapy [file 12885_2020_6882_MOESM2_ESM.docx]

**Table S1. Evidence for antibiotic susceptibility of microbes related to immunotherapy response**

| **Organism** | **Cephalosporins** | **Piperacillin-Tazobactam** | **Amoxicillin-Clavulanic acid** | **Trimethoprim-Sulfamethoxazole** | **Tetracyclines** | **Vancomycin** | **Clindamycin** | **Fluoroquinolones (ciprofloxacin)** | **Macrolides** | **Metronidazole** | **Linezolid (oxazolidinone)** |
| --- | --- | --- | --- | --- | --- | --- | --- | --- | --- | --- | --- |
| Bacteroides clarus (Bacteroidales) | No (1–3) | Yes (4,5) | Yes (1,6) | No (7) | Yes (8) | No (6) | Yes (6) | Yes (9,10) | Yes (11) | Yes (6,12,13) | Yes (14) |
| Prevotella histicola (Bacteroidales) | No (1–3) | Yes (4,5) | Yes (1,6) | No (7) | Yes (8,15) | No (6) | Yes (6) | Yes (9,10) | Yes (11) | Yes (6,12,13) | Yes (14) |
| Parabacteroides distasonis (Bacteroidales) | No (1–3) | Yes (4,5) | Yes (1,6) | No (7) | Yes (8,15) | No (6) | Yes (6) | Yes (9,10) | Yes (11) | Yes (6,12,13) | Yes (14) |
| Bacteroides nordii (Bacteroides) | No (1–3) | Yes (4,5) | Yes (1,6) | No (7) | Yes (8,15) | No (6) | Yes (6) | Yes (9,10) | Yes (11) | Yes (6,12,13) | Yes (14) |
| Porphyromonas pasteri (Bacteroidales) | No (1–3) | Yes (4,5) | Yes (1,6) | No (7) | Yes (8,15) | No (6) | Yes (6) | Yes (9,10) | Yes (11) | Yes (6,12,13) | Yes (14) |
| Akkermansia muciniphila (Verrucomicrobia) | Yes (5) | Yes (5) | Yes (16) | No (7) | Yes (15) | No (17,18) | Yes (19) | No (18) | Yes (11) | No (13) | Yes (14) |
| Desulfovibrio alaskensis (Desulfovibrio spp.) | Yes (20) | Yes (5,20) | No (20) | No (2) | No (20) | Yes (20) | No (20,21) | Yes (22) | No (20) | Yes (20,22) | Yes (14,23) |
| Bifidobacterium longum (Actinobacteria) | Yes (2,3,24) | Yes (5) | Yes (13) | No (7,25) | Yes (15) | Yes (25) | Yes (24) | Yes (25) | Yes (25) | No (24) | Yes (14) |
| Bifidobacterium adolescentis (Actinobacteria) | Yes (2,3,24) | Yes (5) | Yes (13) | No (7,25) | Yes (15) | Yes (25) | Yes (24) | Yes (25) | Yes (25) | No (24) | Yes (14) |
| Gardnerella vaginalis (Actinobacteria) | Yes (2,3,24) | Yes (5) | Yes (13) | No (7,25) | Yes (15) | Yes (25) | Yes (24) | Yes (25) | Yes (25) | No (24) | Yes (14) |
| Collinsella aerofaciens (Actinobacteria) | Yes (2,3,24) | Yes (5) | Yes (13) | No (7,25) | Yes (15) | Yes(25) | Yes (24) | Yes (25) | Yes (25) | No (24) | Yes (14) |
| Blautia obeum (Clostridiales) | No (2,5) | Yes (5,26) | Yes (27) | No (7,26) | Yes (15) | Yes (26) | No (26)^43^ | Yes (9,13) | No (11) | Yes (12,26) | Yes (14) |
| Clostridium bolteae (Clostridiales) | No (2,5) | Yes (5,26) | Yes (27) | No (7,26) | Yes (15) | Yes (26) | No (26) | Yes (9,13) | No (11) | Yes (9,13) | Yes (14) |
| Roseburia intestinalis (Clostridiales) | No (2,5) | Yes (5,26) | Yes (27) | No (7,26) | Yes (15) | Yes (26) | No (26) | Yes (9,13) | No (11) | Yes (9,13) | Yes (14) |
| Ruminococcus bromii (Clostridiales) | No (2,5) | Yes (5,26) | Yes (27) | No (7,26) | Yes (15) | Yes (26) | No (26) | Yes (9,13) | No (11) | Yes (9,13) | Yes (14) |
| Clostridium hungatei (Clostridiales) | No (2,5) | Yes (5,26) | Yes (27) | No (7,26) | Yes (15) | Yes (26) | No (26) | Yes (9,13) | No (11) | Yes (9,13) | Yes (14) |
| Cloacibacillis porcorum (Synergistales) | Yes (28) | Yes (5) | Yes (28) | Yes (29) | Yes (15,28,29) | No (28,29) | Yes (28) | Yes (10,29) | Yes (29) | Yes (28,29) | Yes (14,23) |
| Klebsiella pneumoniae (Enterobacteriales) | No (3) | No (3) | Yes (30) | Yes (24,31) | Yes (8,15,32) | No (23) | Yes (33) | Yes (9,30,34) | Yes (15) | No (23) | No (23) |

1. Nakano V, do Nascimento e Silva A, Merino VRC, Wexler HM, Avila-Campos MJ. Antimicrobial resistance and prevalence of resistance genes in intestinal Bacteroidales strains. Clinics (Sao Paulo). 2011 Apr;66(4):543–7.

2. Sutter VL, Finegold SM. Susceptibility of Anaerobic Bacteria to 23 Antimicrobial Agents. 1976;10:17.

3. Endimiani A, Perez F, Bonomo RA. Cefepime: a reappraisal in an era of increasing antimicrobial resistance. Expert Rev Anti Infect Ther. 2008 Dec;6(6):805–24.

4. Valeria dos Santos K, Diniz CG, Coutinho SC, Apolonio ACM, Geralda de Sousa-Gaia L, Nicoli JR, et al. In vitro activity of piperacillin/tazobactam and ertapenem against Bacteroides fragilis and Escherichia coli in pure and mixed cultures. Journal of Medical Microbiology. 2007 Jun 1;56(6):798–802.

5. Shono Y, Docampo MD, Peled JU, Perobelli SM, Velardi E, Tsai JJ, et al. Increased GVHD-related mortality with broad-spectrum antibiotic use after allogeneic hematopoietic stem cell transplantation in human patients and mice. Sci Transl Med. 2016 May 18;8(339):339ra71.

6. Song YL, Liu CX, McTeague M, Finegold SM. “Bacteroides nordii” sp. nov. and “Bacteroides salyersae” sp. nov. Isolated from Clinical Specimens of Human Intestinal Origin. J Clin Microbiol. 2004 Dec;42(12):5565–70.

7. Smilack JD. Trimethoprim-Sulfamethoxazole. Mayo Clinic Proceedings. 1999 Jul;74(7):730–4.

8. Chopra I, Roberts M. Tetracycline Antibiotics: Mode of Action, Applications, Molecular Biology, and Epidemiology of Bacterial Resistance. Microbiol Mol Biol Rev. 2001 Jun;65(2):232–60.

9. Grillon A, Schramm F, Kleinberg M, Jehl F. Comparative Activity of Ciprofloxacin, Levofloxacin and Moxifloxacin against Klebsiella pneumoniae, Pseudomonas aeruginosa and Stenotrophomonas maltophilia Assessed by Minimum Inhibitory Concentrations and Time-Kill Studies. Nguyen MH, editor. PLoS ONE. 2016 Jun 3;11(6):e0156690.

10. Berlanga M, Palau M, Guerrero R. Gut microbiota dynamics and functionality in Reticulitermes grassei after a 7-day dietary shift and ciprofloxacin treatment. Wilson BA, editor. PLoS ONE. 2018 Dec 27;13(12):e0209789.

11. Suchodolski JS, Dowd SE, Westermarck E, Steiner JM, Wolcott RD, Spillmann T, et al. The effect of the macrolide antibiotic tylosin on microbial diversity in the canine small intestine as demonstrated by massive parallel 16S rRNA gene sequencing. BMC Microbiol. 2009;9(1):210.

12. Willis AT. Metronidazole in the prevention and treatment of bacteroides infections in surgical patients. In: Brumfitt W, Hamilton-Miller JMT, editors. New perspectives in clinical microbiology [Internet]. Dordrecht: Springer Netherlands; 1978 [cited 2019 Aug 16]. p. 47–62. (New perspectives in clinical microbiology). Available from: https://doi.org/10.1007/978-94-011-7508-1_4

13. Giau VV, Wu SY, Jamerlan A, An SSA, Kim SY, Hulme J. Gut Microbiota and Their Neuroinflammatory Implications in Alzheimer’s Disease. Nutrients. 2018 Nov 14;10(11).

14. Jones RN, Johnson DM, Erwin ME. In vitro antimicrobial activities and spectra of U-100592 and U-100766, two novel fluorinated oxazolidinones. Antimicrob Agents Chemother. 1996 Mar;40(3):720–6.

15. Zuckerman JM, Qamar F, Bono BR. Macrolides, Ketolides, and Glycylcyclines: Azithromycin, Clarithromycin, Telithromycin, Tigecycline. Infectious Disease Clinics of North America. 2009 Dec;23(4):997–1026.

16. Dubourg G, Cornu F, Edouard S, Battaini A, Tsimaratos M, Raoult D. First isolation of Akkermansia muciniphila in a blood-culture sample. Clinical Microbiology and Infection. 2017 Sep 1;23(9):682–3.

17. Dubourg G, Lagier J-C, Armougom F, Robert C, Audoly G, Papazian L, et al. High-level colonisation of the human gut by Verrucomicrobia following broad-spectrum antibiotic treatment. International Journal of Antimicrobial Agents. 2013 Feb 1;41(2):149–55.

18. Schell MA, Karmirantzou M, Snel B, Vilanova D, Berger B, Pessi G, et al. The genome sequence of Bifidobacterium longum reflects its adaptation to the human gastrointestinal tract. Proc Natl Acad Sci U S A. 2002 Oct 29;99(22):14422–7.

19. Behr C, Ramírez-Hincapié S, Cameron HJ, Strauss V, Walk T, Herold M, et al. Impact of lincosamides antibiotics on the composition of the rat gut microbiota and the metabolite profile of plasma and feces. Toxicol Lett. 2018 Oct 15;296:139–51.

20. Dzierzewicz Z, Cwalina B, Jaworska-Kik M, Weglarz L, Wilczok T. Susceptibility to antibiotics and biochemical properties of Desulfovibrio desulfuricans strains. Acta Pol Pharm. 2001 Dec;58(6):439–45.

21. Feio MJ, Zinkevich V, Beech IB, Llobet-Brossa E, Eaton P, Schmitt J, et al. Desulfovibrio alaskensis sp. nov., a sulphate-reducing bacterium from a soured oil reservoir. Int J Syst Evol Microbiol. 2004 Sep;54(Pt 5):1747–52.

22. Goldstein EJC, Citron DM, Peraino VA, Cross SA. Desulfovibrio desulfuricans Bacteremia and Review of Human Desulfovibrio Infections. J Clin Microbiol. 2003 Jun;41(6):2752–4.

23. Sweeney MT, Zurenko GE. In Vitro Activities of Linezolid Combined with Other Antimicrobial Agents against Staphylococci, Enterococci, Pneumococci, and Selected Gram-Negative Organisms. Antimicrobial Agents and Chemotherapy. 2003 Jun 1;47(6):1902–6.

24. Butta H, Sardana R, Vaishya R, Singh KN, Mendiratta L. Bifidobacterium: An Emerging Clinically Significant Metronidazole-resistant Anaerobe of Mixed Pyogenic Infections. Cureus. 2017 Apr 4;9(4):e1134.

25. Masco L, Van Hoorde K, De Brandt E, Swings J, Huys G. Antimicrobial susceptibility of Bifidobacterium strains from humans, animals and probiotic products. J Antimicrob Chemother. 2006 Jul 1;58(1):85–94.

26. Schuetz AN. Antimicrobial Resistance and Susceptibility Testing of Anaerobic Bacteria. Clinical Infectious Diseases. 2014 Sep 1;59(5):698–705.

27. Akhavan BJ, Vijhani P. Amoxicillin. In: StatPearls [Internet]. Treasure Island (FL): StatPearls Publishing; 2019 [cited 2019 Jul 11]. Available from: http://www.ncbi.nlm.nih.gov/books/NBK482250/

28. Downes J, Vartoukian SR, Dewhirst FE, Izard J, Chen T, Yu W-H, et al. Pyramidobacter piscolens gen. nov., sp. nov., a member of the phylum ‘Synergistetes’ isolated from the human oral cavity. Int J Syst Evol Microbiol. 2009 May;59(Pt 5):972–80.

29. Vartoukian SR, Downes J, Palmer RM, Wade WG. Fretibacterium fastidiosum gen. nov., sp. nov., isolated from the human oral cavity. International Journal of Systematic and Evolutionary Microbiology. 2013;63(2):458–63.

30. Hawser SP, Bouchillon SK, Lascols C, Hackel M, Hoban DJ, Badal RE, et al. Susceptibility of Klebsiella pneumoniae Isolates from Intra-Abdominal Infections and Molecular Characterization of Ertapenem-Resistant Isolates▿. Antimicrob Agents Chemother. 2011 Aug;55(8):3917–21.

31. Gleckman R, Blagg N, Joubert DW. Trimethoprim: mechanisms of action, antimicrobial activity, bacterial resistance, pharmacokinetics, adverse reactions, and therapeutic indications. Pharmacotherapy. 1981 Aug;1(1):14–20.

32. El Nekidy WS, Mooty MY, Attallah N, Cardona L, Bonilla MF, Ghazi IM. Successful treatment of multidrug resistant Klebsiella pneumoniae using dual carbapenem regimen in immunocompromised patient. IDCases. 2017 Jun 15;9:53–5.

33. Baltch AL, Smith RP, Hammer MC, Conroy JV, Michelsen PB. Antimicrobial effect of clindamycin in combination with aztreonam or aminoglycosides against *Klebsiella* spp. J Antimicrob Chemother. 1991;27(3):303–10.

34. Sader HS, Biedenbach DJ, Jones RN. Global patterns of susceptibility for 21 commonly utilized antimicrobial agents tested against 48,440 Enterobacteriaceae in the SENTRY Antimicrobial Surveillance Program (1997-2001). Diagnostic Microbiology and Infectious Disease. 2003 Sep;47(1):361–4.
